# Functional alteration of divided attention in people living with HIV based on a task-fMRI study

**DOI:** 10.3389/fnins.2025.1667360

**Published:** 2026-01-16

**Authors:** Junzhuo Chen, Zhongtian Guan, Chuanke Hou, Xingyuan Jiang, Haixia Luo, Fan Xu, Aixin Li, Xi Wang, Wei Wang, Chunlin Li, Hongjun Li

**Affiliations:** 1 Department of Radiology, Beijing Youan Hospital, Capital Medical University, Beijing, China; 2 School of Biomedical Engineering, Capital Medical University, Beijing, China; 3 STD/AIDS Clinic, Department of Infectious Diseases, Beijing Youan Hospital, Capital Medical University, Beijing, China

**Keywords:** audio-visual stimulation, divided attention, functional magnetic resonance imaging (fMRI), people living with HIV (PLWH), spatiotemporal task

## Abstract

**Background:**

Impaired attention is a key feature of HIV-associated brain damage, and people living with HIV (PLWH) often have potential visual–auditory perceptual deficits. This study aimed to explore functional alterations in divided attention in PLWH using a parallel audio-visual spatiotemporal task with multimodal functional magnetic resonance imaging (fMRI) and to explore candidate neuroimaging markers of HIV-related attention impairment.

**Methods:**

Thirty-one cognitively unimpaired PLWH and 34 healthy controls (HC) completed a divided attention task during fMRI via a modified Posner paradigm. Behavioral performance and task-related brain activation were compared between the two groups. Seed-based whole-brain functional connectivity (FC) maps were computed in resting-state fMRI (rs-fMRI) using *a priori* anatomical regions of interest (ROIs) from the audiovisual attention network, defined based on previous independent fMRI studies employing similar spatial–temporal attention paradigms.

**Results:**

The PLWH showed lower accuracy than HC. Task-related brain activation was more extensive in PLWH, including increased activation in occipital/temporal lobes, plus frontal/parietal lobes, insula, and limbic system. Using *a priori* anatomical regions of interest from the audiovisual attention network as seeds, PLWH exhibited increased resting-state FC between these frontal–parietal–temporal–insular regions and bilateral posterior cerebellar lobules VIII–IX, as well as with multimodal associative cortices. Within the PLWH group, percent BOLD signal change showed significant positive correlations with HIV infection duration in a subset of task-difference ROIs—7 regions identified under spatial cueing and 13 regions identified under temporal cueing.

**Conclusion:**

The HIV impairs audio-visual divided attention, with fMRI revealing neural alterations in cognitively unimpaired PLWH. These findings suggest that task-related activation patterns and resting-state connectivity measures may serve as sensitive candidate markers of HIV-related brain involvement and help identify individuals at increased risk of cognitive decline, although longitudinal studies are needed to establish their prognostic value.

## 1 Introduction

Human immunodeficiency virus (HIV) is neurotropic and can cross the blood–brain barrier early in infection, causing neuronal damage through direct and indirect mechanisms (Sami Saribas et al., 2017; Saylor et al., 2016). This may lead to HIV-associated neurocognitive disorders (HAND), characterized by cognitive, behavioral, motor, and autonomic dysfunction (Heaton et al., 2010). Although combined antiretroviral therapy (cART) effectively suppresses viral replication, it fails to eliminate latent viral reservoirs in the brain (Rojas-Celis et al., 2019; Wallet et al., 2019). HIV-related brain damage significantly impairs patients’ quality of life. In the pre-cART era, motor delay and slowed processing speed predominated, whereas attention deficits, learning/memory impairment, and executive dysfunction now represent the primary cognitive manifestations (Lew et al., 2018; Sacktor and Robertson, 2014; Saylor et al., 2016).

Impaired attention is a key feature of HIV-associated brain damage and one of the earliest affected neurocognitive domains (Wang et al., 2017). As a core component of information processing, attention enables selective stimulus prioritization and behavioral optimization, thereby enhancing overall cognitive efficiency (He et al., 2024). It also serves as a foundational cognitive function, supporting other domains such as executive control and behavioral flexibility (Arif et al., 2020; Squire et al., 2013). Clinically, attentional deficits significantly impact daily functioning, for instance, impairing driving performance and treatment adherence. However, the neural mechanisms underlying attention dysfunction in people living with HIV (PLWH) remain poorly understood (Wang et al., 2017).

While traditional neuropsychological tests have been used to assess attention function, their subjective nature and lack of direct correlation with neural changes limit their sensitivity. Notably, neurobehavioral impairments often precede detectable cognitive symptoms among asymptomatic PLWH (Lew et al., 2018), highlighting the need for more objective biomarkers to identify early neural damage. Neuroimaging techniques, particularly functional magnetic resonance imaging (fMRI), offer a valuable non-invasive approach: fMRI enables monitoring of brain activity and functional connectivity (FC) during tasks or at rest, and has revealed altered activity patterns and FC in multiple brain regions among PLWH (Hall et al., 2021; Jiang et al., 2025; Liu et al., 2020), making it a promising tool to address this need.

Additionally, several studies have shown that PLWH have potential impairments of visual and auditory perception (Han et al., 2022; Heilman et al., 2013; Li et al., 2019; Torre et al., 2015). Visual and auditory information processing is critical for higher-order brain functions (e.g., learning, memory, language, and information processing). In natural environments, attentional resources are allocated across sensory modalities to process information; when receiving simultaneous multi-sensory stimuli (e.g., visual and auditory), the distribution of attention across these modalities is termed “divided attention” (Wang et al., 2022). Notably, audio-visual attentional processing operates in both spatial and temporal domains, representing higher-level cognitive processes distinct from basic sensory functions (Allman et al., 2014; Macaluso et al., 2003; Moore and Zirnsak, 2017). Spatial and temporal attention interact dynamically, integrating “what,” “where,” and “when” information (Nobre and Van Ede, 2018; Tang et al., 2013). While previous fMRI studies have examined unimodal sensory deficits in PLWH, none have investigated how HIV affects spatial and temporal attention in parallel audiovisual systems. This study integrates these dimensions to comprehensively explore HIV-associated divided attention deficits and to identify potential neuroimaging candidate markers of HIV-related brain involvement, aiming to inform targeted interventions and improve prognosis. To this end, we employed an audiovisual divided-attention task based on a modified Posner cueing framework that manipulates endogenous, top-down attention along both spatial (“where”) and temporal (“when”) dimensions. This paradigm was derived from previously validated fMRI tasks of visually cued auditory spatial and temporal attention (Li et al., 2012; Wang et al., 2022; Zhang et al., 2025) and was further adapted from a two-cue EEG version (Tang et al., 2013) for use in the MRI environment by retaining endogenous spatial and temporal cues while simplifying the design for a clinical PLWH population.

## 2 Materials and methods

### 2.1 Participants

This study adhered to the Declaration of Helsinki and was approved by the Ethics Committee of Beijing Youan Hospital. All participants provided written informed consent. In line with national epidemiological data showing a clear male predominance among Chinese adults living with HIV in the 20–45-year age range (Hou et al., 2023; Wei et al., 2025; Zhao et al., 2020), we restricted the sample to right-handed men aged 20–45 years. Thirty-five PLWH were recruited from the STD/AIDS Clinic of Beijing Youan Hospital, and 35 healthy controls (HC) matched for age and education level (all HC were also male) were recruited from the community.

The inclusion criteria were as follows: (1) no contraindications for MR examination; (2) normal vision and hearing; (3) no history of substance abuse (alcohol/drugs); (4) no history of neurological disorders (e.g., stroke, traumatic brain injury, brain tumors); and (5) no history of psychiatric diseases. ELISA, Western blot, or PCR confirmed HIV infection. Four PLWH and one HC were excluded due to incomplete or poor-quality MR images, resulting in a final sample of 31 PLWH and 34 HC. Demographic and clinical information was obtained from participants’ self-reports and electronic medical records, including age, educational level, duration of HIV infection, treatment duration, nadir and current plasma CD4^+^ counts, CD4^+^/CD8^+^ ratio, and plasma viral load. All PLWH acquired HIV infection via male-to-male sexual contact. Outpatient follow-up records and self-reported medication histories indicated generally good treatment adherence: no participant had interrupted cART in the 6 months before MR examination, and plasma HIV RNA levels at the most recent follow-up were undetectable in all PLWH. With respect to treatment regimen, 21 patients were receiving DTG/FTC/TAF (dolutegravir + emtricitabine + tenofovir alafenamide), 7 were receiving TDF/3TC/EFV (tenofovir disoproxil fumarate + lamivudine + efavirenz), and 3 were receiving TAF/FTC/EVG (tenofovir alafenamide + emtricitabine + elvitegravir).

### 2.2 Neurocognitive tests

All participants completed the neurocognitive tests to assess cognitive status. The tests included the following six cognitive domains: (1) Verbal and language: Animal verbal fluency test (AFT); (2) Attention/working memory: Wechsler memory scale-III (WMS-III) and Paced auditory serial addition test (PASAT); (3) Memory (learning and recall): Hopkins verbal learning test (HVLT-R) and Brief visuospatial memory test (BVMT-R); (4) Speed of information processing: Trail marking test A (TMT-A); (5) Fine motor skills: Grooved Pegboard; (6) Abstraction/executive: Wisconsin card sorting tests-64 (WCST-64).

The raw scores of each test were converted into *T*-scores using validated norm data from the Chinese population (mean = 50, standard deviation = 10). The norms were sourced from Shi et al. (2015) and adjusted for factors such as age, gender, years of education, and the population size of the city where the participants grew up. For cognitive domains including multiple tests, the average of the *T*-scores of these tests was calculated as the final score of that domain.

According to the Frascati criteria (Antinori et al., 2007), HAND can be diagnosed if the final score of a cognitive domain is more than one standard deviation below the aforementioned T-score norm (i.e., *T*-score ≤ 40) in at least two of the six cognitive domains.

### 2.3 Task-fMRI design

The visual stimuli were generated using the Psychtoolbox platform in MATLAB 2022b (Mathworks, Natick, MA, United States), and presented via an MR-compatible audio-visual system. Participants were equipped with noise-canceling earmuffs to attenuate the noise. Custom MR-compatible buttons were used. Before the start of the MR scan, each subject received a detailed task explanation and completed a practice session. Reaction time and accuracy were recorded during formal scanning.

Participants performed an audiovisual divided-attention task based on a modified Posner cueing paradigm with spatial and temporal visual cues (Li et al., 2012; Posner et al., 1980; Tang et al., 2013; Wang et al., 2022; Zhang et al., 2025). Each trial began with a central fixation cross presented for 1,500 ms, followed by a 100-ms central visual cue indicating either the attended spatial location or the attended temporal interval. In the spatial cue condition, one side of a central diamond was thickened, pointing left or right to indicate the visual field in which the upcoming target was most likely to appear. In the temporal cue condition, the inner or outer ring of a central concentric circle was thickened, signaling a short (300 ms) or long (1,500 ms) cue–target interval, respectively. After the cue–target interval (300 or 1,500 ms), a white visual target (“×”) was presented for 50 ms in the left or right peripheral box, synchronized with a 50-ms burst of lateralized white noise delivered to the left or right ear. Participants were instructed to attend to both modalities and to press the left or right button only when the visual and auditory targets appeared on the same side (congruent trials), and to withhold responses when the two stimuli appeared on opposite sides (incongruent trials). A 1200-ms response window followed the target onset.

**Figure 1:**
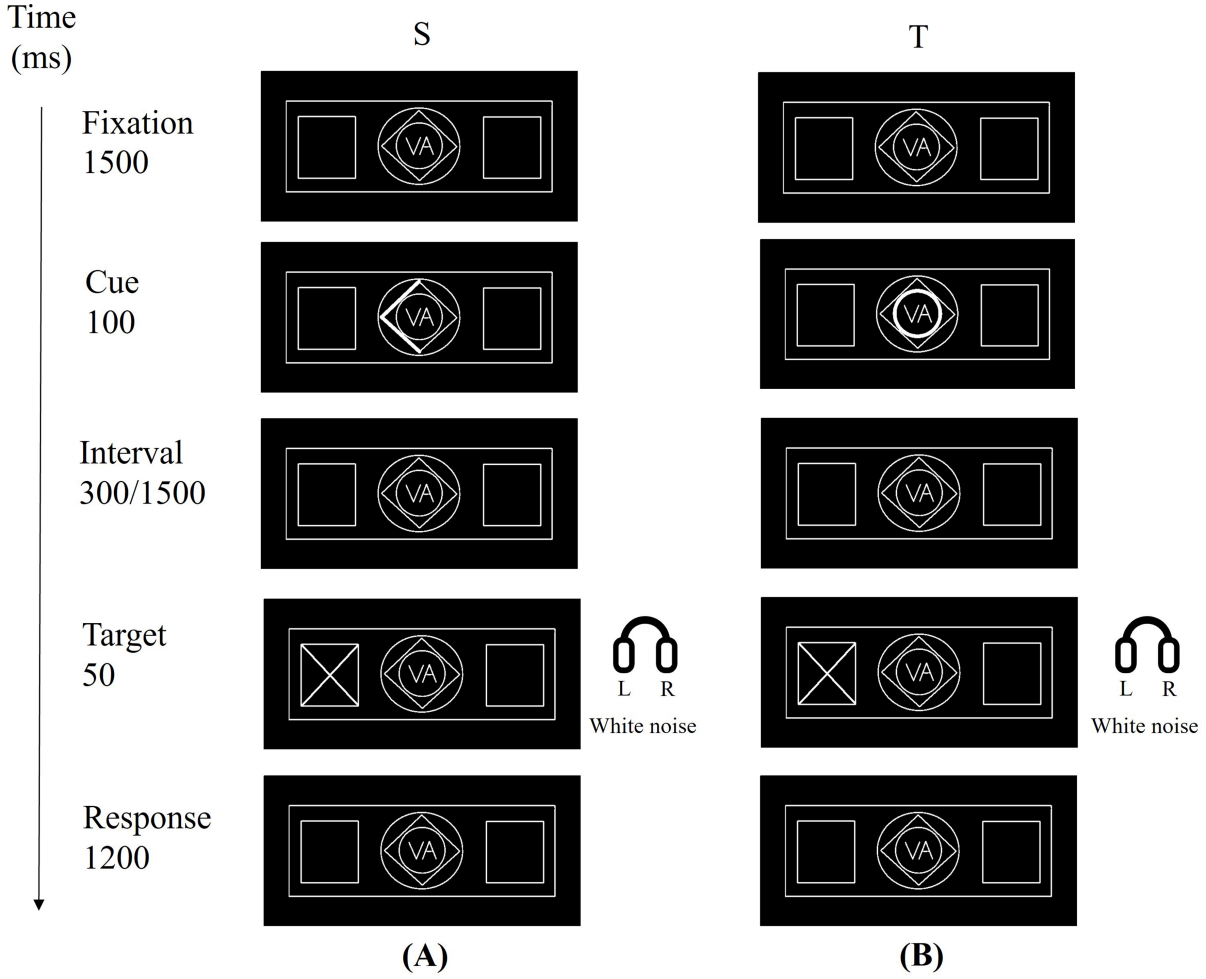
Task experimentation paradigm. (First row) Fixation (1,500 ms). (Second row) Cue presentation: **(A)** S, Spatial cue: arrow indicates target location (left/right); **(B)** T, Temporal cue: inner/outer circle indicates short (300 ms) or long (1,500 ms) interval. (Third row) Interval period (300 ms or 1,500 ms). (Fourth row) Target presentation: a visual stimulus (“×”) appeared in the left/right box for 50 ms, accompanied by a 50-ms white noise (auditory stimulus) played in the left/right ear simultaneously. (Fifth row) Response phase (1,200 ms): participants pressed the left/right button to report the target location if audio-visual stimuli were on the same side (no response required for mismatched locations).

Each participant completed two task-fMRI runs. Each run contained 60 trials (30 spatially cued and 30 temporally cued), organized into six pseudo-random blocks of 10 trials. Within each run, left versus right target locations, short versus long cue–target intervals, and congruent versus incongruent audiovisual combinations were approximately balanced, with valid cues comprising about 80% of all trials and invalid cues about 20% in both cue conditions. The two runs had identical numbers of trials and conditions but independent pseudo-randomized trial sequences (cue type, target side, interval, congruency). The first 10 volumes of each run were discarded to allow for signal stabilization. Each run lasted approximately 4 min, with a brief (~20 s) rest between runs, yielding a total task-fMRI scan time of about 9 min. The overall trial sequence is illustrated in Figure 1.

### 2.4 MRI data acquisition

MRI data were acquired on a 3.0T MR scanner (GE SIGNA Pioneer, America) using a 32-channel head coil. T1-weighted structural images were acquired using the following magnetization-prepared rapid gradient-echo sequence: repetition time (TR) = 7.8 ms, echo time (TE) = 3.2 ms, acquisition matrix = 256 × 256, flip angle = 8°, and voxel resolution = 1 mm x 1 mm x 1 mm, number of layers = 188, slice thickness = 1 mm. Functional imaging was conducted using the following gradient-echo single-shot echo planar imaging (EPI) sequence: TR = 2,000 ms, TE = 30 ms, acquisition matrix = 64 × 64, voxel size = 3.5 mm x 3.5 mm x 3.5 mm, flip angle = 90°, number of layers = 36 (axial direction), slice thickness = 3.5 mm, inter-slice gap = 0.5 mm.

### 2.5 MRI data processing

#### 2.5.1 fMRI preprocessing

For the fMRI data, we first used MRIcro^1^ to convert the DICOM files to NIFTI files. Preprocessing steps were performed with SPM12 (Wellcome Department of Cognitive Neurology, London, United Kingdom) implemented in MATLAB 2022b. The preprocessing steps included: (1) removing the first 10 time points to reduce the instability of the magnetic field; (2) taking the scanning time of the middle layer as a reference, aligning the time of all layers with that of the middle layer; (3) realigning the correction for head motion and excluding subjects whose head moved more than 2 mm or rotated more than 2°; (4) normalizing the above-obtained images to the Montreal Neurological Institute (MNI) space and resampling them to a voxel size of 3 × 3 × 3 mm^3^; (5) smoothing the normalized images with an isotropic 8 × 8 × 8 full-width at half-maximum Gaussian kernel.

#### 2.5.2 Brain activation analysis

The first-stage analysis used a general linear model (GLM) to estimate task-activation patterns for each attention condition, with blood-oxygen-level-dependent (BOLD) responses modeled as neural activity convolved with a canonical hemodynamic response function (HRF). Nuisance regressors included six head-motion parameters, and low-frequency noise was removed using a 128-s high-pass filter. Post-estimation, contrasts for each task generated individual brain activation maps. Second-stage analysis compared group differences in activation patterns between PLWH and HC using independent-samples t-tests, with age and education as covariates. Statistical thresholds were set at voxel-wise *p* < 0.001 and cluster-wise *p* < 0.05 (false discovery rate [FDR] corrected).

#### 2.5.3 Seed-based whole-brain FC analysis

Using DPABI V4.2 software (Yan et al., 2016), we conducted seed-based rs-fMRI analyses using *a priori* anatomical regions of interest (ROIs). Specifically, based on previous independent fMRI studies employing similar audiovisual spatial–temporal attention tasks (Li et al., 2012; Wang et al., 2022; Zhang et al., 2025), we selected six bilateral pairs of ROIs (12 ROIs in total) from the AAL atlas corresponding to core nodes of the audiovisual attention network: middle frontal gyrus/dorsolateral prefrontal cortex (MFG/DLPFC), supplementary motor area (SMA), insula (INS), inferior frontal gyrus (IFG), inferior parietal lobule/supramarginal gyrus (IPL/SMG), and superior temporal/temporo-parietal regions (STG/TPJ). For each ROI, the mean resting-state time series was extracted and correlated with voxel-wise time series across the whole brain to generate seed-to-voxel FC maps. Group differences in FC between PLWH and HC were assessed using independent-samples *t*-tests with age and education as covariates. Statistical significance was set at voxel-wise *p* < 0.001 and cluster-level *p* < 0.05 (FDR corrected).

#### 2.5.4 Correlation analyses

For each attention condition (spatial and temporal), we first defined task-difference regions of interest (ROIs) as clusters showing significant group differences in task-related activation at the whole-brain level. For each task-difference ROI, we then calculated the percent BOLD signal change during the attention task relative to the pre-cue fixation baseline. Using SPSS 27.0 (IBM Corp., Armonk, NY, United States), Pearson correlation analyses were performed within the PLWH group to examine associations between ROI-wise percent signal change and clinical variables (current CD4^+^ T cell count, CD4^+^/CD8^+^ ratio, plasma viral load, infection duration, treatment duration, and nadir CD4^+^ T cell count). Statistical significance was set at two-tailed *p* < 0.05 with FDR correction for multiple comparisons.

### 2.6 Statistical analyses

Group differences in age, education, neurocognitive test scores, accuracy, and reaction time were analyzed using SPSS 27.0. Independent-samples *t*-tests or Mann–Whitney *U* tests were applied as appropriate, with statistical significance set at two-tailed *p* < 0.05.

## 3 Results

### 3.1 Demographic, clinical, and neuropsychological data

The demographic data, clinical characteristics, and neurocognitive test results are presented in Table 1. In the PLWH group, the mean duration of HIV infection was 3.94 ± 3.02 years, and the mean duration of cART was 2.98 ± 2.08 years (Table 1). There were no significant demographic differences between the two groups. According to the Frascati criteria (Antinori et al., 2007), none of the 31 PLWH and 34 HC included in this study had cognitive impairment.

**Table 1:** Demographic, clinical, and neuropsychological data.

Variables	PLWH (*n* = 31)	HC (*n* = 34)	*p*-value
Age (years)	30.29 ± 3.56	31.09 ± 3.48	0.365^a^
Education level (years)	15(12–19)	16(13–20)	0.410^b^
Duration of infection (years)	3.94 ± 3.02	N/A	N/A
Duration of treatment (years)	2.98 ± 2.08	N/A	N/A
Current CD4^+^ count (cells/mL)	629.15 ± 197.83	N/A	N/A
Current CD4^+^/CD8^+^ ratio	0.82 ± 0.41	N/A	N/A
Nadir CD4^+^ count (cells/mL)	362.41 ± 112.36	N/A	N/A
Current viral load	TND	N/A	N/A
Scores of cognitive performances			
Speed of information processing	45.34 ± 3.97	46.62 ± 4.74	0.245^a^
Memory (learning and recall)	44.27 ± 4.19	48.37 ± 5.00	<0.001^a^
Verbal and language	44.54 ± 3.15	49.09 ± 4.81	<0.001^a^
Abstraction/executive	45.65 ± 4.85	48.41 ± 4.66	0.022^a^
Fine motor skills	43.84 ± 2.86	47.06 ± 3.58	<0.001^a^
Attention/working memory	43.15 ± 2.92	48.87 ± 5.09	<0.001^a^

Normally distributed variables are listed as mean ± SD, while non-normally distributed variables are listed as median (IQR). Significance was set as *p* < 0.05, two-tailed. The two groups’ clinical data and neurocognitive test scores were compared using independent-samples *t*-tests or the Mann–Whitney *U* test. ^a^*t*-test; ^b^Mann–Whitney *U* test. HC, healthy control; PLWH, people living with HIV; TND, virus not detectable; N/A, not available; SD, standard deviation.

### 3.2 Behavioral performance

**Figure 2:**
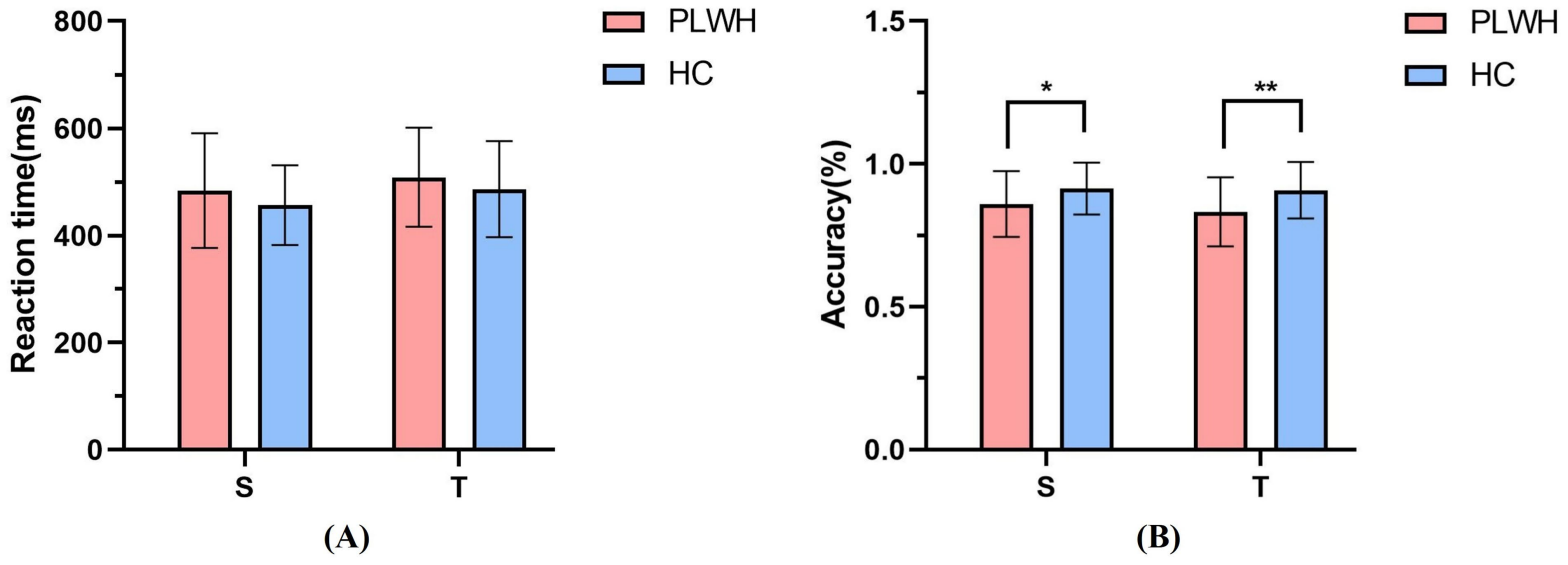
Behavioral manifestations. **(A)** Average reaction time. **(B)** Average accuracy rate (S, spatial attention condition; T, temporal attention condition; *p < 0.05, **p < 0.01).

Reaction times (RTs) from incorrect or missed trials, as well as RTs faster than 100 ms or slower than 1,000 ms, were excluded from the RT analyses. No trials in either group had RTs outside this 100–1,000 ms range. For PLWH, mean RTs in the spatial and temporal cue conditions were 457.16 ± 76.44 ms and 508.75 ± 92.55 ms, respectively; for HC, mean RTs in the two conditions were 479.87 ± 102.52 ms and 486.34 ± 89.58 ms, respectively (Figure 2A). Thus, in both groups, mean RTs were numerically faster in the spatial than in the temporal cue condition.

The mean accuracy (ACC) of PLWH in the spatial and temporal conditions was 85.97% ± 11.56 and 83.24% ± 12.07%, respectively. The mean ACC of HC in the two conditions was 91.36% ± 9.10 and 90.82% ± 9.85%. Group differences in ACC across the two cue conditions were significant (spatial: *p* = 0.044; temporal: *p* = 0.009), with HC showing higher accuracy than PLWH in both conditions (Figure 2B). To further characterize behavioral performance, we also quantified the number and proportion of excluded trials. In HC, there were 107 incorrect trials (5.25%) and 62 missed trials (3.04%) in the spatial cue condition, and 111 incorrect trials (5.44%) and 76 missed trials (3.73%) in the temporal cue condition. In PLWH, there were 156 incorrect trials (8.39%) and 107 missed trials (5.75%) in the spatial cue condition, and 192 incorrect trials (10.32%) and 115 missed trials (6.18%) in the temporal cue condition. Although exclusion rates were somewhat higher in PLWH, consistent with their lower overall accuracy, the proportion of excluded trials remained modest in both groups (all <17%), and all RT and accuracy analyses were conducted on the remaining valid trials.

### 3.3 Group differences in brain activation patterns

**Figure 3:**
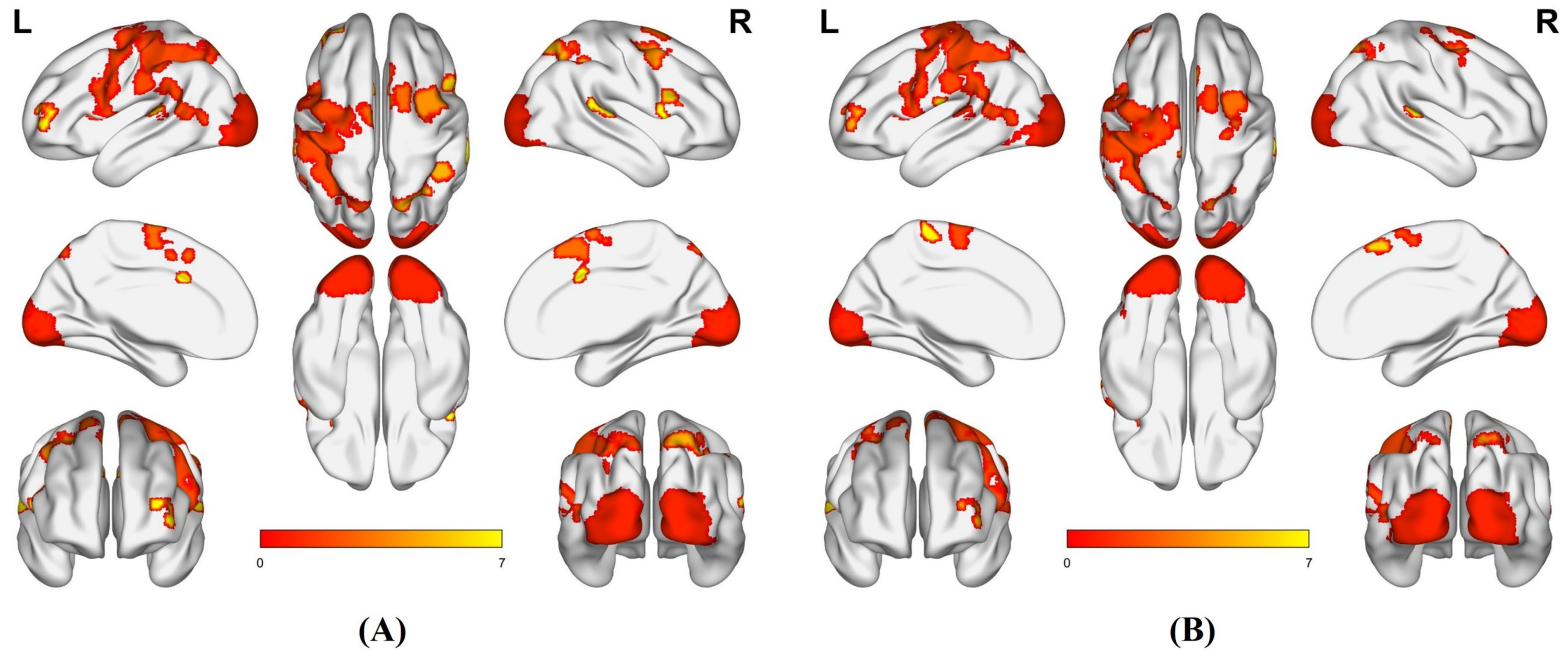
Brain regions with increased activation in PLWH vs. HC. **(A)** Spatial attention condition; **(B)** Temporal attention condition (L, left hemisphere; R, right hemisphere).

Compared with HC, PLWH showed enhanced activation in both conditions in the left lingual gyrus (LING. L), left rolandic operculum (ROL. L), left superior temporal gyrus (STG. L), and right middle frontal gyrus (MFG. R). In the spatial condition specifically, PLWH exhibited additional enhanced activation in the bilateral insula (INS. R/L), right triangular part of the inferior frontal gyrus (IFGtriang. R), and right medial/paracingulate gyrus (DCG. R). In the temporal condition, unique enhanced activation was observed in the left postcentral gyrus (PoCG. L), left paracentral lobule (PCL. L), and right angular gyrus (ANG. R). Statistical thresholds were set at voxel-wise *p* < 0.001 and cluster-wise *p* < 0.05 (FDR corrected) (Figure 3 and Tables 2, 3).

**Table 2:** Increased activation in PLWH vs. HC (spatial condition).

Brain region	Hemisphere	Peak MNI coordinates			Cluster size (voxels)	*T*-value
		*X* (mm)	*Y* (mm)	*Z* (mm)		
LING	L	−20	−96	−18	6,446	1.24
INS	R	42	18	−2	72	10.03
ORBmid	L	−42	50	−2	226	7.28
ROL	L	−58	8	0	5,549	2.14
INS	L	−32	14	7	50	12.01
MTG	L	−56	−32	6	177	8.06
IFGtriang	R	58	20	6	51	11.15
STG	R	60	−30	8	76	9.37
IFGoperc	R	48	16	16	349	6.12
IPL	R	42	−54	38	539	5.43
MFG	R	38	2	40	623	4.29
DCG	R	10	10	44	823	3.31

Coordinates (*X*, *Y*, *Z*) refer to the peak MNI (Montreal Neurological Institute) coordinates of brain regions with peak intensity (LING, lingual; INS, insula; ORBmid, middle frontal gyrus, orbital part; ROL, rolandic operculum; MTG, middle temporal gyrus; IFGtriang, inferior frontal gyrus, triangular part; STG, superior temporal gyrus; IFGoperc, inferior frontal gyrus, opercular part; IPL, inferior parietal lobule; MFG, middle frontal gyrus; DCG, median cingulate and paracingulate gyri; L, left hemisphere; R, right hemisphere).

**Table 3:** Increased activation in PLWH vs. HC (temporal condition).

Brain region	Hemisphere	Peak MNI coordinates			Cluster size (voxels)	*T*-value
		*X* (mm)	*Y* (mm)	*Z* (mm)		
LING	L	−20	−96	−18	6,349	1.22
ORBmid	L	−42	50	−2	164	5.01
ROL	L	−60	8	0	5,983	2.46
PoCG	L	−60	−14	14	74	7.31
PCL	L	−4	−28	60	54	10.05
STG	R	60	−30	8	47	11.26
IFGoperc	R	52	14	32	66	8.53
MFG	R	38	2	42	423	3.25
ANG	R	32	−62	48	193	4.33
SMA	R	4	16	52	78	6.25

Coordinates (*X*, *Y*, *Z*) refer to the peak MNI (Montreal Neurological Institute) coordinates of brain regions with peak intensity (LING, lingual; ORBmid, middle frontal gyrus, orbital part; ROL, rolandic operculum; PoCG, postcentral gyrus; PCL, paracentral lobule; STG, superior temporal gyrus; IFGoperc, inferior frontal gyrus, opercular part; MFG, middle frontal gyrus; ANG, angular gyrus; SMA, supplementary motor area; L, left hemisphere; R, right hemisphere).

### 3.4 The whole-brain FC based on seed regions

**Figure 4:**
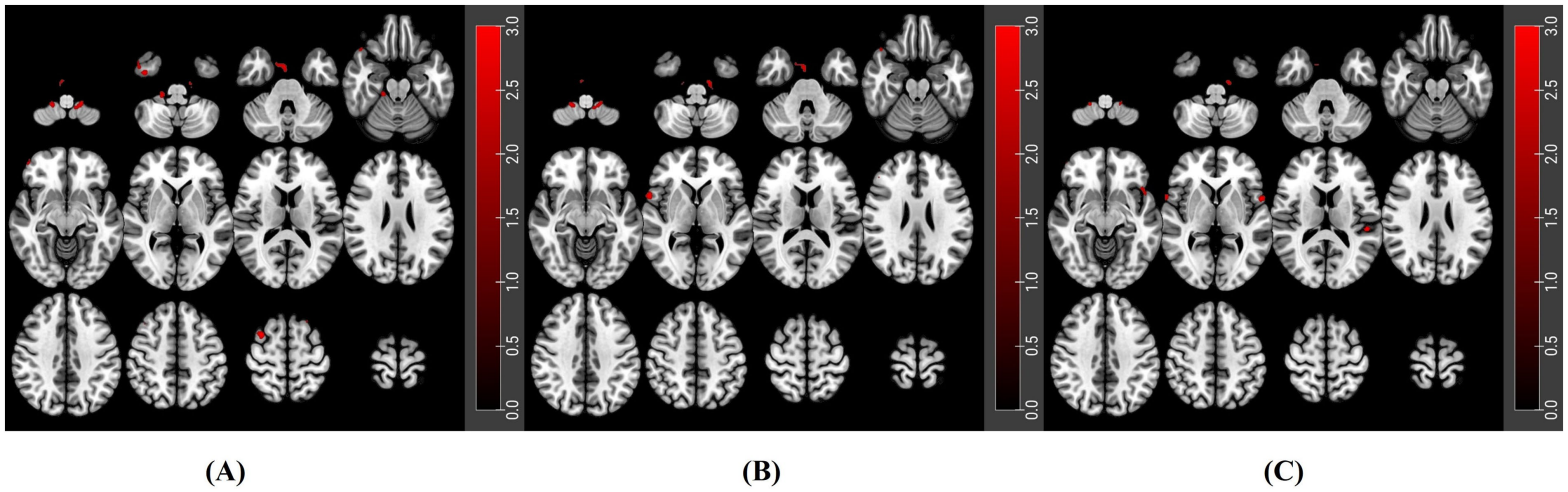
Seed-based resting-state FC differences between PLWH and HC for three representative *a priori* ROIs. **(A)** MFG. L, left middle frontal gyrus. **(B)** SMG. R, right supramarginal gyrus; **(C)** STG. L, left superior temporal gyrus.

Using the 12 *a priori* anatomical ROIs described above, PLWH showed a highly convergent pattern of enhanced resting-state FC compared with HC. Across most seeds, PLWH exhibited significantly increased connectivity with bilateral cerebellar lobules VIII–IX (Cerebelum_8/9. L/R). In addition, several seeds showed stronger FC with multimodal associative cortices, including the inferior frontal gyrus, inferior temporal gyrus, supramarginal gyrus, and Rolandic operculum. Statistical thresholds were set at voxel-wise *p* < 0.001 and cluster-wise *p* < 0.05 (FDR corrected). Representative seed-based connectivity maps for the left MFG, right SMG, and left STG are shown in Figure 4, and connectivity maps for the following 9 ROIs are provided in the Supplementary Figure 1. The detailed results of FC between all 12 a priori ROI brain regions and the whole brain are presented in Supplementary Table 1.

### 3.5 Correlation analysis results

**Figure 5:**
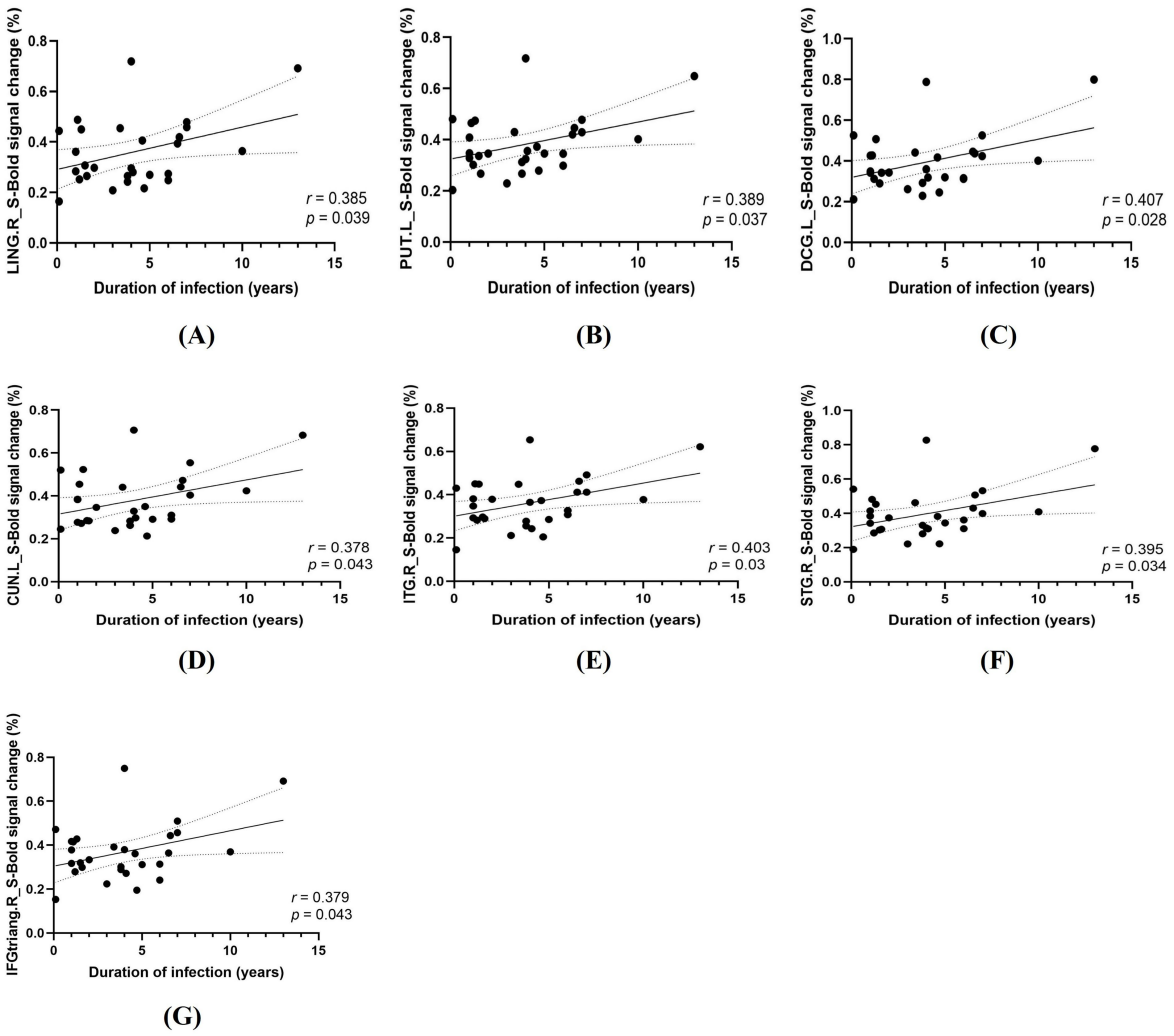
Correlations between HIV infection duration and task-related activation in the spatial cueing condition (S). **(A)** LING. R, right lingual gyrus. **(B)** PUT. L, left putamen. **(C)** DCG. L, left median cingulate/paracingulate gyri; **(D)** CUN. L, left cuneus; **(E)** ITG. R, right inferior temporal gyrus; **(F)** STG. R, right superior temporal gyrus; **(G)** IFGtriang. R, right triangular part of the inferior frontal gyrus.

**Figure 6:**
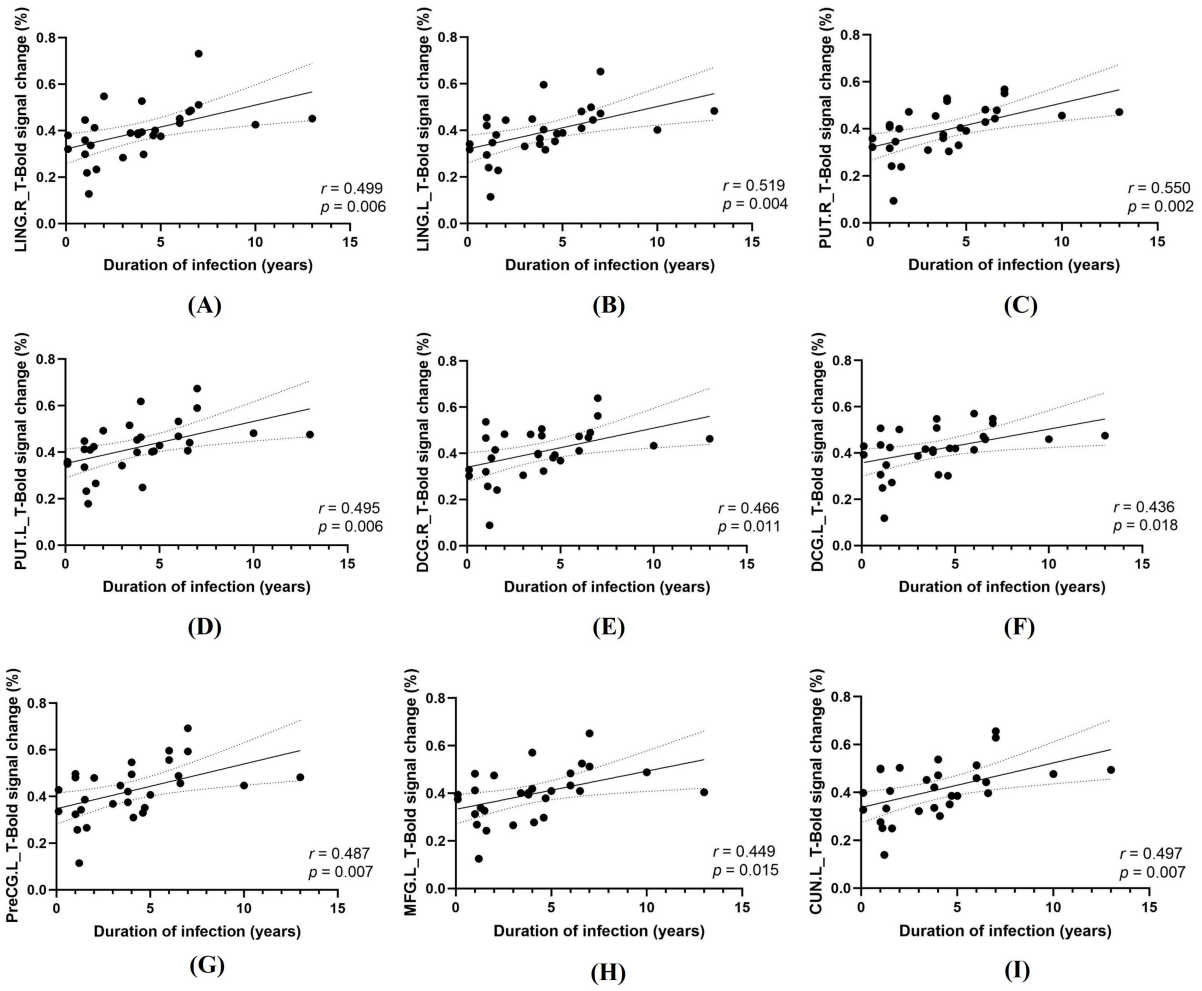
Correlations between HIV infection duration and task-related activation in the temporal cueing condition (T). **(A)** LING. R, right lingual gyrus; **(B)** LING. L, left lingual gyrus; **(C)** PUT. R, right putamen; **(D)** PUT. L, left putamen; **(E)** DCG. R, right median cingulate/paracingulate gyri; **(F)** DCG. L, left median cingulate/paracingulate gyri; **(G)** PreCG. L, left precentral gyrus; **(H)** MFG. L, left middle frontal gyrus; **(I)** CUN. L, left cuneus.

In PLWH, Pearson correlation analyses revealed significant positive associations between HIV infection duration and task-related percent BOLD signal change in 7 task-difference ROIs under spatial cueing (all surviving FDR correction; Figure 5) and 13 task-difference ROIs under temporal cueing (all surviving FDR correction; 9 ROIs displayed in Figure 6 and 4 additional ROIs presented in Supplementary Figure 2). By contrast, no significant correlations were observed between activation in these ROIs and current CD4^+^ T cell count, CD4^+^/CD8^+^ ratio, plasma viral load, treatment duration, or nadir CD4^+^ T cell count after FDR correction (all *p* > 0.05).

## 4 Discussion

Cross-modal attention and multisensory integration are essential for world perception (Li et al., 2015). This study is the first to employ a parallel audio-visual spatiotemporal task under fMRI to investigate divided attention deficits in PLWH. The results showed that PLWH exhibit preclinical behavioral impairments in divided attention and altered brain function, indicating that fMRI may be more sensitive than traditional neurocognitive tests to subtle brain functional changes and may provide candidate imaging markers for flagging HIV-associated brain involvement before overt cognitive decline; however, longitudinal studies are required to confirm their predictive value.

In task performance, PLWH had significantly lower accuracy than HC under both attention conditions, with no group differences in reaction times. At the same time, both groups, particularly the HC group, responded numerically faster and more accurately to spatial cues than to temporal cues. This indicates that the paradigm elicited the expected facilitatory effects of spatial cueing on behavioral performance. This behavioral pattern is consistent with previous Posner-type audiovisual attention studies, in which spatial cueing has been shown to confer an advantage in both speed and accuracy (Li et al., 2012; Tang et al., 2013; Wang et al., 2022; Zhang et al., 2025). This pattern indicates impaired cross-modal attention resource allocation rather than deficits in basic sensory processing speed. Previous studies suggest that timing tasks are more sensitive to early cognitive decline than traditional neurocognitive tests (Hinkin et al., 2000), making these accuracy reductions potential markers of HIV-related cognitive impairment. The dual-task paradigm requires dynamic allocation of limited attentional resources across visual and auditory modalities, processes compromised in PLWH due to pre-attentive system disruption (Agmon et al., 2022; Hinkin et al., 2000). Notably, PLWH performed normally on standard neurocognitive tests, likely because single-task demands did not exceed their compensatory capacity. In contrast, the audio-visual dual task revealed their inability to meet the higher demands of divided attention.

The PLWH showed impairments in both temporal and spatial orientation, requiring greater and more extensive brain activation to complete the same task under each attention condition. Attention modulates multisensory integration with interactions between stimulus types (Yan et al., 2015): visual–spatial cues orient attention to locations, and visual–temporal cues to time intervals, with partially overlapping but dissociable neural mechanisms (Tang et al., 2013). In the present study, PLWH showed increased activation in occipital visual regions (e.g., lingual gyrus and cuneus), temporal auditory/multimodal cortices (e.g., superior and inferior temporal gyri), and frontal and parietal association areas (e.g., middle frontal gyrus and inferior parietal/supramarginal regions) under both spatial and temporal cueing. This constellation of regions closely corresponds to the canonical dorsal frontoparietal attention network and associated occipital visual cortices that support goal-directed orienting to spatial locations and time intervals, as well as ventral/salience-network regions (including anterior insula and inferior frontal cortex) that detect behaviorally relevant events and reorient attention (Corbetta and Shulman, 2002; Coull and Nobre, 1998; Macaluso et al., 2003; Moore and Zirnsak, 2017; Nobre and Van Ede, 2018). In line with these models, our findings suggest that cognitively unimpaired PLWH rely more heavily on these fronto-parietal–occipital systems to sustain divided audiovisual attention, which may reflect compensatory recruitment in the context of early HIV-related neural alterations.

While neural networks for spatial and temporal orientation overlap substantially, condition-specific activation increases in certain regions indicate functional specialization. In the spatial condition, PLWH showed heightened activation in INS. R/L, IFGtriang. R, and DCG. R. INS and DCG, as core salience network components, mediate stimulus detection, filtering, and orientation (Fu et al., 2020; Mengotti et al., 2022; Zhou and Seeley, 2014). Cross-modal divided attention requires greater spatial salience precision, prompting PLWH to mobilize reserves for more extensive salience network activation. INS also regulates detection of salient stimuli and task-oriented cognitive control (Marwood et al., 2018; Mundy, 2018; Sheffield et al., 2020); increased insular ReHo in PLWH may reflect adaptive cognitive feedback, correlating positively with disease duration (Han et al., 2022). IFGtriang. R, linked to task-switching and multitasking (aiding attention shifts), also shows reliable differences between PLWH and HC in rs-fMRI (Plessis et al., 2014).

Temporal attention describes how directing resources to specific time points influences behavior (Coull and Nobre, 1998), providing a temporal reference for action initiation. It is impaired in many diseases, e.g., vigilance network dysfunction in mesial temporal lobe epilepsy (Englot et al., 2020). In the temporal condition, PLWH showed heightened activation in PoCG. L, PCL. L, and ANG. R. PoCG and PCL interact with temporal attention in cognitive tasks; their collaboration supports complex processing (e.g., dynamic visual/auditory) via spatiotemporal integration to optimize perception and responses. Melrose (2008) similarly noted increased PoCG activation in PLWH during integration tasks.

In the resting-state analyses, seed-based FC using the 12 *a priori* anatomical ROIs from the audiovisual attention network revealed a convergent pattern of increased connectivity in PLWH compared with HC, predominantly involving the canonical fronto-parietal–occipital attention systems and their cerebellar extensions. Across most seeds, PLWH showed significantly stronger coupling between fronto-parietal–temporal–insular attention regions and bilateral posterior cerebellar lobules VIII–IX (Cerebelum_8/9. L/R), together with increased FC to multimodal associative cortices such as the inferior frontal gyrus, inferior temporal gyrus, supramarginal gyrus, and Rolandic operculum. This pattern is consistent with prior work demonstrating that large portions of the human cerebellum are functionally connected with cerebral association networks and that posterior cerebellar regions form an integral part of the dorsal frontoparietal attention network and other control networks involved in visuospatial orienting (Brissenden et al., 2016; Buckner, 2013). More broadly, the observation that inferior frontal and parietal associative regions show enhanced coupling with these cerebellar territories aligns with canonical models in which dorsal frontoparietal and ventral attention networks jointly support goal-directed and stimulus-driven attentional control (Corbetta and Shulman, 2002; Nobre and Van Ede, 2018). Taken together, these findings extend previous reports of altered large-scale connectivity in PLWH by emphasizing additional involvement of cerebellar and multimodal associative regions, and raise the possibility that enhanced cortico–cerebellar coupling may reflect compensatory reorganization in cognitively unimpaired PLWH. Nonetheless, longitudinal studies will be required to determine whether such connectivity increases are maintained, normalized, or eventually give way to connectivity reductions as the disease progresses.

These findings imply compensatory neuroplasticity in PLWH, with heightened brain activation reflecting attempted functional preservation despite HIV-induced neural damage (Toniolo et al., 2020). The mechanism behind HIV-related attention deficits remains unclear, but altered activation/connectivity may involve brain inflammation. HIV activates monocytes to invade the CNS, releasing inflammatory factors (e.g., TNF-α, IL-1β) and neurotoxic proteins (e.g., gp120, Tat, Vpr) that disrupt homeostasis and impair function (Ellis et al., 2023; Saylor et al., 2016). PET study (Vera et al., 2016) further identifies elevated transporter protein expression in PLWH, particularly in parietal-occipital and globus pallidus regions, linking neuroinflammation to cognitive decline.

Chang et al. (2004) demonstrated that greater HIV disease severity (lower CD4^+^ counts, higher viral loads) correlated with increased BOLD signals during visual attention tasks. Similarly, EEG studies revealed larger P200 amplitudes in PLWH during attention tasks, showing significant associations with infection duration (Meghdadi et al., 2021). Our findings further support these observations. These findings demonstrate that neuropathological progression correlates closely with HIV infection duration, likely mediated by persistent cerebral viral replication and neuroinflammation. Sustained intracerebral HIV replication alters metabolism, impacting BOLD responsiveness (Roc et al., 2007). Chronic infection drives progressive functional damage through persistent neuroinflammation and neuroglial activation (Ferguson et al., 2016; Gendelman and Gelbard, 2014). Notably, BOLD changes did not correlate with CD4^+^ counts or viral load, likely due to universal cART use. While cART controls plasma virus (mostly undetectable) and CD4^+^ levels, many agents poorly cross the blood–brain barrier, leaving the brain as an HIV reservoir. Systemic treatment does not eliminate intracerebral viral replication or neuroinflammation, so PLWH may experience ongoing virus-mediated neural damage despite normal plasma markers (Ellero et al., 2017; Whitehead et al., 2014).

At present, HAND assessment relies on neurocognitive tests, subjective with poor sensitivity. The neuroimaging of PLWH showed abnormalities before neurocognitive tests, indicating a decline in neural efficiency. If the load exceeds the brain’s reserve capacity, it may lead to cognitive impairment (Chang et al., 2004; Tomasi et al., 2006). This explains persistent cognitive deficits in PLWH despite cART-controlled virus. With more long-lived PLWH, early detection of subclinical damage and targeted interventions are key to reducing HAND (Ernst et al., 2009). Recently, non-invasive therapies like computer-based cognitive rehabilitation therapy (CRT) and transcranial magnetic stimulation (TMS) show promise, particularly for attention/working memory in patients with better virological control (Begemann et al., 2020; Wei et al., 2022; Zondo, 2023). fMRI-guided targeting may optimize these interventions by identifying vulnerable neural networks, potentially preventing HAND progression.

## 5 Limitations and prospects

This study has some limitations. First, it was a single-center study with a relatively small sample size. Second, all participants in the present study were right-handed men aged 20–45 years. Although this sampling strategy is consistent with the male predominance among Chinese adults living with HIV in this age range, it further restricts the applicability of our results to female PLWH. Third, all PLWH included in this study were treatment-experienced, virally suppressed individuals on long-term cART. Antiretroviral therapy itself, as well as differences between specific regimens, may influence neuroimaging measures; therefore, our findings are best interpreted as reflecting brain functional characteristics in treated, virally suppressed PLWH with good adherence rather than untreated HIV infection. Finally, the cross-sectional design limits our ability to delineate the temporal trajectory of HIV-related brain changes. Future multicenter studies with larger and more diverse samples, including female PLWH and patients with different treatment histories, and longitudinal follow-up designs will be needed to more comprehensively characterize the course and treatment-related modulation of HIV-associated brain damage.

## 6 Conclusion

While cART has reduced HAND incidence, it remains a significant HIV complication, impairing quality of life and treatment adherence. Our study demonstrates HIV-induced audiovisual divided attention impairment, characterized by altered brain activity and FC preceding measurable cognitive deficits. fMRI offers sensitive biomarkers to objectively identify patients at risk of developing HAND, enabling early targeted interventions to improve prognosis.

## Data Availability

The original contributions presented in the study are included in the article/Supplementary material, further inquiries can be directed to the corresponding authors.

## Footnotes

1
http://www.cabiatl.com/mricro/

## References

[ref1] Agmon G. Yahav P. H.-S. Ben-Shachar M. Golumbic E. Z. (2022). Attention to speech: mapping distributed and selective attention systems. Cereb. Cortex 32, 3763–3776. doi: 10.1093/cercor/bhab446, 34875678

[ref3] Allman M. J. Teki S. Griffiths T. D. Meck W. H. (2014). Properties of the internal clock: first- and second-order principles of subjective time. Annu. Rev. Psychol. 65, 743–771. doi: 10.1146/annurev-psych-010213-115117, 24050187

[ref4] Antinori A. Arendt G. Becker J. T. Brew B. J. Byrd D. A. Cherner M. . (2007). Updated research nosology for HIV-associated neurocognitive disorders. Neurology 69, 1789–1799. doi: 10.1212/01.WNL.0000287431.88658.8b, 17914061 PMC4472366

[ref5] Arif Y. Wiesman A. I. O’Neill J. Embury C. May P. E. Lew B. J. . (2020). The age-related trajectory of visual attention neural function is altered in adults living with HIV: a cross-sectional MEG study. EBioMedicine 61:103065. doi: 10.1016/j.ebiom.2020.103065 33099087 PMC7585051

[ref6] Begemann M. J. Brand B. A. Ćurčić-Blake B. Aleman A. Sommer I. E. (2020). Efficacy of non-invasive brain stimulation on cognitive functioning in brain disorders: a meta-analysis. Psychol. Med. 50, 2465–2486. doi: 10.1017/S0033291720003670, 33070785 PMC7737055

[ref7] Brissenden J. A. Levin E. J. Osher D. E. Halko M. A. Somers D. C. (2016). Functional evidence for a cerebellar node of the dorsal attention network. J. Neurosci. 36, 6083–6096. doi: 10.1523/JNEUROSCI.0344-16.2016, 27251628 PMC4887569

[ref8] Buckner R. L. (2013). The cerebellum and cognitive function: 25 years of insight from anatomy and neuroimaging. Neuron 80, 807–815. doi: 10.1016/j.neuron.2013.10.044, 24183029

[ref9] Chang L. Tomasi D. Yakupov R. Lozar C. Arnold S. Caparelli E. . (2004). Adaptation of the attention network in human immunodeficiency virus brain injury. Ann. Neurol. 56, 259–272. doi: 10.1002/ana.20190, 15293278

[ref10] Corbetta M. Shulman G. L. (2002). Control of goal-directed and stimulus-driven attention in the brain. Nat. Rev. Neurosci. 3, 201–215. doi: 10.1038/nrn755, 11994752

[ref11] Coull J. T. Nobre A. C. (1998). Where and when to pay attention: the neural systems for directing attention to spatial locations and to time intervals as revealed by both PET and fMRI. J. Neurosci. 18, 7426–7435. doi: 10.1523/JNEUROSCI.18-18-07426.1998, 9736662 PMC6793260

[ref12] Ellero J. Lubomski M. Brew B. (2017). Interventions for neurocognitive dysfunction. Curr. HIV/AIDS Rep. 14, 8–16. doi: 10.1007/s11904-017-0346-z, 28110422

[ref13] Ellis R. J. Marquine M. J. Kaul M. Fields J. A. Schluachetzki J. C. M. (2023). Mechanisms underlying HIV-associated cognitive impairment and emerging therapies for its management. Nat. Rev. Neurol. 19, 668–687. doi: 10.1038/s41582-023-00879-y, 37816937 PMC11052664

[ref14] Englot D. J. Morgan V. L. Chang C. (2020). Impaired vigilance networks in temporal lobe epilepsy: mechanisms and clinical implications. Epilepsia 61, 189–202. doi: 10.1111/epi.16423, 31901182 PMC7033006

[ref15] Ernst T. Yakupov R. Nakama H. Crocket G. Cole M. Watters M. . (2009). Declined neural efficiency in cognitively stable human immunodeficiency virus patients. Ann. Neurol. 65, 316–325. doi: 10.1002/ana.21594, 19334060 PMC2734503

[ref16] Ferguson D. Clarke S. Berry N. Almond N. (2016). Attenuated SIV causes persisting neuroinflammation in the absence of a chronic viral load and neurotoxic antiretroviral therapy. AIDS 30, 2439–2448. doi: 10.1097/QAD.0000000000001178, 27258396 PMC5051525

[ref17] Fu D. Weber C. Yang G. Kerzel M. Nan W. Barros P. . (2020). What can computational models learn from human selective attention? A review from an audiovisual unimodal and Crossmodal perspective. Front. Integr. Neurosci. 14:10. doi: 10.3389/fnint.2020.00010, 32174816 PMC7056875

[ref18] Gendelman H. E. Gelbard H. A. (2014). Adjunctive and long-acting nanoformulated antiretroviral therapies for HIV-associated neurocognitive disorders. Curr. Opin. HIV AIDS 9, 585–590. doi: 10.1097/COH.0000000000000111, 25226025 PMC4231201

[ref19] Hall S. A. Towe S. L. Nadeem M. T. Hobkirk A. L. Hartley B. W. Li R. . (2021). Hypoactivation in the precuneus and posterior cingulate cortex during ambiguous decision making in individuals with HIV. J. Neurovirol. 27, 463–475. doi: 10.1007/s13365-021-00981-1, 33983505 PMC8276275

[ref20] Han S. Aili X. Ma J. Liu J. Wang W. Yang X. . (2022). Altered regional homogeneity and functional connectivity of brain activity in young HIV-infected patients with asymptomatic neurocognitive impairment. Front. Neurol. 13:982520. doi: 10.3389/fneur.2022.982520, 36303561 PMC9593212

[ref21] He C. Yuan T. Yuan L. Wang J. Lu X. Hu W. . (2024). Selective attention function impairment in HIV-negative patients with early forms of neurosyphilis. Eur. J. Med. Res. 29:408. doi: 10.1186/s40001-024-02004-1, 39113099 PMC11304781

[ref23] Heaton R. K. Clifford D. B. Franklin D. R. Woods S. P. Ake C. Vaida F. . (2010). HIV-associated neurocognitive disorders persist in the era of potent antiretroviral therapy: CHARTER study. Neurology 75, 2087–2096. doi: 10.1212/WNL.0b013e318200d727, 21135382 PMC2995535

[ref24] Heilman K. J. Harden E. R. Weber K. M. Cohen M. Porges S. W. (2013). Atypical autonomic regulation, auditory processing, and affect recognition in women with HIV. Biol. Psychol. 94, 143–151. doi: 10.1016/j.biopsycho.2013.06.003, 23792136 PMC3742727

[ref25] Hinkin C. H. Castellon S. A. Hardy D. J. (2000). Dual task performance in HIV-1 infection. J. Clin. Exp. Neuropsychol. 22, 16–24. doi: 10.1076/1380-3395(200002)22:1;1-8;FT016, 10649542

[ref26] Hou Y. Jin Y. Cai C. Qin Q. Tang H. Lyu F. . (2023). Comparative analysis of epidemiological features of HIV/AIDS cases aged over and under 50 years old — China, 2010–2022. China CDC Wkly 5, 1079–1083. doi: 10.46234/ccdcw2023.202, 38058988 PMC10696226

[ref27] Jiang X. Hou C. Ma J. Li H. (2025). Alterations in local activity and whole-brain functional connectivity in human immunodeficiency virus-associated neurocognitive disorders: a resting-state functional magnetic resonance imaging study. Quant. Imaging Med. Surg. 15, 563–580. doi: 10.21037/qims-24-1342, 39838977 PMC11744116

[ref28] Lew B. J. McDermott T. J. Wiesman A. I. O’Neill J. Mills M. S. Robertson K. R. . (2018). Neural dynamics of selective attention deficits in HIV-associated neurocognitive disorder. Neurology 91, e1860–e1869. doi: 10.1212/WNL.0000000000006504 30333162 PMC6260195

[ref29] Li C. Chen K. Han H. Chui D. Wu J. (2012). An fMRI study of the neural systems involved in visually cued auditory top-down spatial and temporal attention. PLoS One 7:e49948. doi: 10.1371/journal.pone.0049948, 23166800 PMC3499497

[ref30] Li Y. Li C. Wu Q. Xu Z. Kurata T. Ohno S. . (2015). Decreased resting-state connections within the visuospatial attention-related network in advanced aging. Neurosci. Lett. 597, 13–18. doi: 10.1016/j.neulet.2015.03.047, 25817360

[ref31] Li R. Wang W. Wang Y. Peters S. Zhang X. Li H. (2019). Effects of early HIV infection and combination antiretroviral therapy on intrinsic brain activity: a cross-sectional resting-state fMRI study. Neuropsychiatr. Dis. Treat. 15, 883–894. doi: 10.2147/NDT.S195562, 31114203 PMC6497505

[ref32] Liu D. Zhao C. Wang W. Wang Y. Li R. Sun J. . (2020). Altered gray matter volume and functional connectivity in human immunodeficiency virus-infected adults. Front. Neurosci. 14:601063. doi: 10.3389/fnins.2020.601063, 33343289 PMC7744568

[ref33] Macaluso E. Eimer M. Frith C. D. Driver J. (2003). Preparatory states in crossmodal spatial attention: spatial specificity and possible control mechanisms. Exp. Brain Res. 149, 62–74. doi: 10.1007/s00221-002-1335-y, 12592504

[ref34] Marwood L. Wise T. Perkins A. M. Cleare A. J. (2018). Meta-analyses of the neural mechanisms and predictors of response to psychotherapy in depression and anxiety. Neurosci. Biobehav. Rev. 95, 61–72. doi: 10.1016/j.neubiorev.2018.09.022, 30278195 PMC6267850

[ref35] Meghdadi A. H. Berka C. Richard C. Rupp G. Smith S. Stevanović Karić M. . (2021). EEG event related potentials in sustained, focused and divided attention tasks: potential biomarkers for cognitive impairment in HIV patients. Clin. Neurophysiol. 132, 598–611. doi: 10.1016/j.clinph.2020.11.026, 33573761 PMC9045835

[ref36] Melrose R. (2008). Compromised fronto-striatal functioning in HIV: an fMRI investigation of semantic event sequencing. Behav. Brain Res. 188, 337–347. doi: 10.1016/j.bbr.2007.11.021, 18242723

[ref37] Mengotti P. Käsbauer A.-S. Fink G. R. Vossel S. (2022). Combined TMS-fMRI reveals behavior-dependent network effects of right temporoparietal junction neurostimulation in an attentional belief updating task. Cereb. Cortex 32, 4698–4714. doi: 10.1093/cercor/bhab511, 35088068

[ref38] Moore T. Zirnsak M. (2017). Neural mechanisms of selective visual attention. Annu. Rev. Psychol. 68, 47–72. doi: 10.1146/annurev-psych-122414-033400, 28051934

[ref39] Mundy P. (2018). A review of joint attention and social-cognitive brain systems in typical development and autism spectrum disorder. Eur. J. Neurosci. 47, 497–514. doi: 10.1111/ejn.13720, 28922520

[ref40] Nobre A. C. Van Ede F. (2018). Anticipated moments: temporal structure in attention. Nat. Rev. Neurosci. 19, 34–48. doi: 10.1038/nrn.2017.141, 29213134

[ref41] Plessis S. D. Vink M. Joska J. A. Koutsilieri E. Stein D. J. Emsley R. (2014). HIV infection and the fronto-striatal system: a systematic review and meta-analysis of fMRI studies. AIDS Lond. Engl. 28, 803–811. doi: 10.1097/QAD.0000000000000151, 24300546

[ref42] Posner M. I. Snyder C. R. Davidson B. J. (1980). Attention and the detection of signals. J. Exp. Psychol. 109, 160–174. doi: 10.1037/0096-3445.109.2.160, 7381367

[ref43] Roc A. C. Ances B. M. Chawla S. Korczykowski M. Wolf R. L. Kolson D. L. . (2007). Detection of human immunodeficiency virus induced inflammation and oxidative stress in lenticular nuclei with magnetic resonance spectroscopy despite antiretroviral therapy. Arch. Neurol. 64, 1249–1257. doi: 10.1001/archneur.64.9.noc60125, 17620480

[ref44] Rojas-Celis V. Valiente-Echeverría F. Soto-Rifo R. Toro-Ascuy D. (2019). New challenges of HIV-1 infection: how HIV-1 attacks and resides in the central nervous system. Cells 8:1245. doi: 10.3390/cells8101245, 31614895 PMC6829584

[ref45] Sacktor N. Robertson K. (2014). Evolving clinical phenotypes in HIV-associated neurocognitive disorders: Curr. Curr Opin. HIV AIDS 9, 517–520. doi: 10.1097/COH.0000000000000102, 25203640 PMC4212639

[ref46] Sami Saribas A. Cicalese S. Ahooyi T. M. Khalili K. Amini S. Sariyer I. K. (2017). HIV-1 Nef is released in extracellular vesicles derived from astrocytes: evidence for Nef-mediated neurotoxicity. Cell Death Dis. 8:e2542–e2542. doi: 10.1038/cddis.2016.467, 28079886 PMC5386374

[ref47] Saylor D. Dickens A. M. Sacktor N. Haughey N. Slusher B. Pletnikov M. . (2016). HIV-associated neurocognitive disorder — pathogenesis and prospects for treatment. Nat. Rev. Neurol. 12, 234–248. doi: 10.1038/nrneurol.2016.27, 26965674 PMC4937456

[ref48] Sheffield J. M. Rogers B. P. Blackford J. U. Heckers S. Woodward N. D. (2020). Insula functional connectivity in schizophrenia. Schizophr. Res. 220, 69–77. doi: 10.1016/j.schres.2020.03.068, 32307263 PMC7322763

[ref49] Shi C. Kang L. Yao S. Ma Y. Li T. Liang Y. . (2015). The MATRICS consensus cognitive battery (MCCB): co-norming and standardization in China. Schizophr. Res. 169, 109–115. doi: 10.1016/j.schres.2015.09.003, 26441005 PMC4916953

[ref50] Squire R. F. Noudoost B. Schafer R. J. Moore T. (2013). Prefrontal contributions to visual selective attention. Annu. Rev. Neurosci. 36, 451–466. doi: 10.1146/annurev-neuro-062111-150439, 23841841

[ref51] Tang X. Li C. Li Q. Gao Y. Yang W. Yang J. . (2013). Modulation of auditory stimulus processing by visual spatial or temporal cue: an event-related potentials study. Neurosci. Lett. 553, 40–45. doi: 10.1016/j.neulet.2013.07.022, 23896527

[ref52] Tomasi D. Chang L. de Castro Caparelli E. Telang F. Ernst T. (2006). The human immunodeficiency virus reduces network capacity: acoustic noise effect. Ann. Neurol. 59, 419–423. doi: 10.1002/ana.20766, 16437575 PMC2440821

[ref53] Toniolo S. Cercignani M. Mora-Peris B. Underwood J. Alagaratnam J. Bozzali M. . (2020). Changes in functional connectivity in people with HIV switching antiretroviral therapy. J. Neurovirol. 26, 754–763. doi: 10.1007/s13365-020-00853-0, 32500477 PMC7532134

[ref54] Torre P. Hoffman H. J. Springer G. Cox C. Young M. A. Margolick J. B. . (2015). Hearing loss among HIV-seropositive and HIV-seronegative men and women. JAMA Otolaryngol. Head Neck Surg. 141:202. doi: 10.1001/jamaoto.2014.3302, 25541676 PMC4369193

[ref55] Vera J. H. Guo Q. Cole J. H. Boasso A. Greathead L. Kelleher P. . (2016). Neuroinflammation in treated HIV-positive individuals: a TSPO PET study. Neurology 86, 1425–1432. doi: 10.1212/WNL.0000000000002485, 26911637 PMC4831035

[ref56] Wallet C. De Rovere M. Van Assche J. Daouad F. De Wit S. Gautier V. . (2019). Microglial cells: the main HIV-1 reservoir in the brain. Front. Cell. Infect. Microbiol. 9:362. doi: 10.3389/fcimb.2019.00362, 31709195 PMC6821723

[ref57] Wang L. Li C. Han Z. Wu Q. Sun L. Zhang X. . (2022). Spatiotemporal and sensory modality attention processing with domain-specific representations in frontoparietal areas. Cereb. Cortex 32, 5489–5502. doi: 10.1093/cercor/bhac029, 35136999

[ref58] Wang Y.-q. Pan Y. Zhu S. Wang Y.-g. Shen Z. Wang K. (2017). Selective impairments of alerting and executive control in HIV-infected patients: evidence from attention network test. Behav. Brain Funct. 13:11. doi: 10.1186/s12993-017-0129-0, 28651626 PMC5485500

[ref59] Wei J. Hou J. Mu T. Sun J. Li S. Wu H. . (2022). Evaluation of computerized cognitive training and cognitive and daily function in patients living with HIV: a meta-analysis. JAMA Netw. Open 5:e220970. doi: 10.1001/jamanetworkopen.2022.0970, 35238931 PMC8895263

[ref60] Wei L. Jia H. Geng Y. Dou Z. Zhao D. Gan X. . (2025). Trends in life expectancy of HIV-infected patients receiving antiretroviral therapy — China, 2013–2023. China CDC Wkly 7, 838–842. doi: 10.46234/ccdcw2025.136, 40620291 PMC12228075

[ref61] Whitehead N. Potterton J. Coovadia A. (2014). The neurodevelopment of HIV-infected infants on HAART compared to HIV-exposed but uninfected infants. AIDS Care 26, 497–504. doi: 10.1080/09540121.2013.841828, 24125015

[ref62] Yan T. Geng Y. Wu J. Li C. (2015). Interactions between multisensory inputs with voluntary spatial attention: an fMRI study. Neuroreport 26, 605–612. doi: 10.1097/WNR.0000000000000368, 26103115

[ref63] Yan C.-G. Wang X.-D. Zuo X.-N. Zang Y.-F. (2016). DPABI: data processing and analysis for (resting-state) brain imaging. Neuroinformatics 14, 339–351. doi: 10.1007/s12021-016-9299-4, 27075850

[ref64] Zhang Z. Guan Z. He M. Liu Y. Yan M. Li C. (2025). Behavioral representations within the endogenous dual attentional pathways during audiovisual integration processing. Front. Neurosci. 19:1536688. doi: 10.3389/fnins.2025.1536688, 40008300 PMC11850320

[ref65] Zhao H. Liu H. Wang L. Yang X. Wang S. Han M. . (2020). Epidemiological characteristics of newly-reported HIV cases among youth aged 15−24 years — China, 2010−2019. China CDC Wkly 2, 913–916. doi: 10.46234/ccdcw2020.249, 34594799 PMC8422364

[ref66] Zhou J. Seeley W. W. (2014). Network dysfunction in Alzheimer’s disease and frontotemporal dementia: implications for psychiatry. Biol. Psychiatry 75, 565–573. doi: 10.1016/j.biopsych.2014.01.020, 24629669

[ref67] Zondo S. (2023). The cognitive remediation of attention in HIV-associated neurocognitive disorders (HAND): a meta-analysis and systematic review. F1000Res 12:1133. doi: 10.12688/f1000research.132166.1, 38778812 PMC11109681

