# Supplementary material for: Functional alteration of divided attention in people living with HIV based on a task-fMRI study
Source: Front Neurosci. 2026 Jan 16;19:1667360. doi: 10.3389/fnins.2025.1667360 (PMC12855078; doi:10.3389/fnins.2025.1667360)
Supplement: Supplementary file 1 [file Data_Sheet_1.pdf]

Supplementary Table 1. Brain regions with enhanced FC with a priori seed ROIs in PLWH vs. HC

Seed region	Brain region	Peak MNI coordinates			Cluster size(voxels)	T value
		X (mm)	Y (mm)	Z (mm)		
MFG.L	Cere9.R	14	-48	-56	391	3.62
	Cere8.R	30	-58	-54	53	2.82
	ITG.R	40	-6	-48	66	3.22
	MFG.R	42	28	44	31	2.99
	SFGdor.R	32	8	62	39	3.55
MFG.R	Cere9.L	12	-48	-56	338	3.71
	Cere8.R	32	-58	-54	63	2.79
SMA.L	Cere9.R	14	-48	-56	79	3.19
	Cere9.L	-16	-46	-58	44	2.75
	ORBinf.R	46	24	-22	28	2.91
SMA.R	Cere9.L	-18	-44	-58	55	2.85
	Cere9.R	14	-48	-56	79	3.19
	IFGoperc.R	58	16	-2	30	2.75
INS.L	Cere9.L	-12	-48	-58	96	2.86
	Cere9.R	12	-48	-56	99	3.48
	IFGtriang.R	54	32	18	58	3.13
INS.R	Cere9.R	12	-48	-56	204	3.64
	IFGoperc.R	58	12	4	61	2.82
	IFGtriang.R	54	32	18	28	2.90
IPL.L	Cere8.R	18	-46	-58	60	3.27
	Cere9.L	-16	-46	-58	33	2.85
IPL.R	Cere9.R	12	-48	-56	305	3.91
SMG.L	Cere9.R	18	-46	-58	76	3.20
	Cere9.L	-16	-46	-58	23	2.65
SMG.R	Cere9.R	12	-48	-56	249	3.64
	Cere8.R	34	-58	-54	41	2.81
	IFGoperc.R	58	10	2	55	2.88
STG.L	Cere8.R	20	-44	-58	39	3.00
	Cere9.L	-18	-44	-58	29	2.65
	TPOsup.L	-50	14	-12	49	3.01
	ROL.L	-56	8	2	20	3.04
STG.R	Cere9.R	14	-48	-56	54	3.26
	Cere9.L	-8	-52	-56	43	2.77
	Cere8.R	42	-56	-56	24	2.78
	ROL.R	60	10	2	76	3.02

Coordinates (X, Y, Z) refer to the peak MNI (Montreal Neurological Institute) coordinates of brain regions with peak intensity (MFG, middle frontal gyrus; SMA, supplementary motor area; INS,

insula; IPL, inferior parietal lobule; SMG, supramarginal gyrus; STG, superior temporal gyrus; L, left hemisphere; R, right hemisphere).

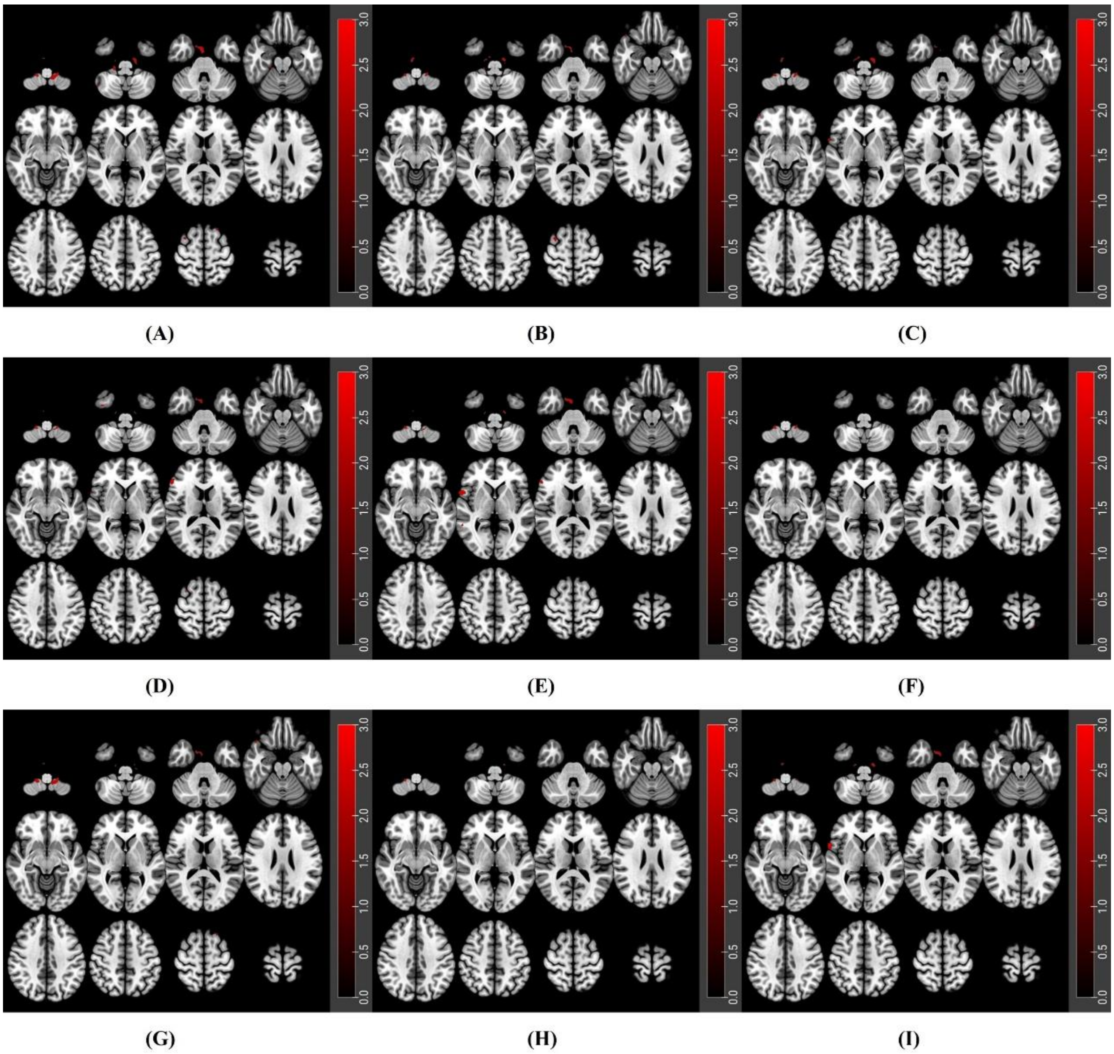

Supplementary Figure 1. Seed-based resting-state functional connectivity differences for the remaining nine a priori ROIs. (A) MFG.R, right middle frontal gyrus; (B) SMA.L, left supplementary motor area; (C) SMA.R, right supplementary motor area; (D) IPL.L, left inferior parietal lobule; (E) IPL.R, right inferior parietal lobule; (F) SMG.L, left supramarginal gyrus; (G) STG.R, right superior temporal gyrus; (H) INS.L, left insula; (I) INS.R, right insula.

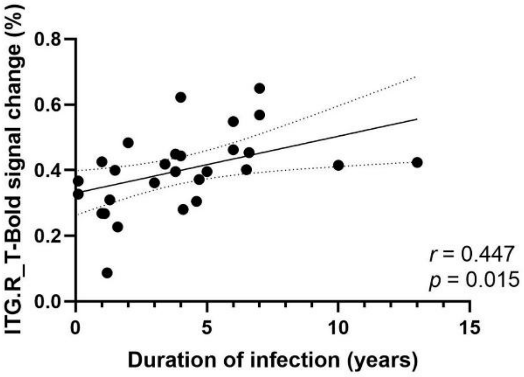

(A)

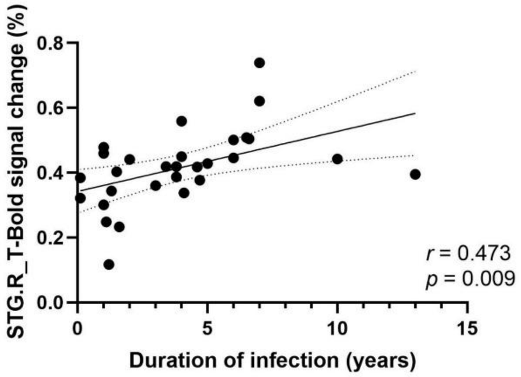

(B)

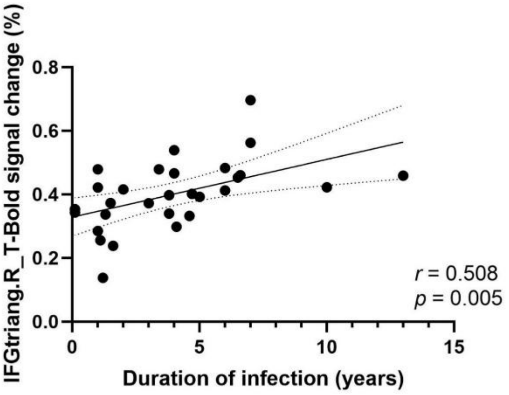

(C)

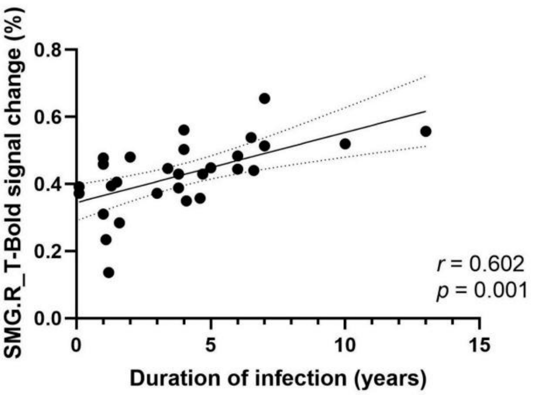

(D)

Supplementary Figure 2. Additional temporal-cueing ROIs showing significant activation–duration correlations. (A) ITG.R, right inferior temporal gyrus; (B) STG.R, right superior temporal gyrus; (C) IFGtriang.R, right triangular part of the inferior frontal gyrus; (D) SMG.R, right supramarginal gyrus.
